# Directed differentiation of hPSCs through a simplified lateral plate mesoderm protocol for generation of articular cartilage progenitors

**DOI:** 10.1371/journal.pone.0280024

**Published:** 2023-01-27

**Authors:** Christopher A. Smith, Evelyn Amelia Humphreys, Mark A. Naven, Steven Woods, Fabrizio E. Mancini, Julieta O’Flaherty, Qing-Jun Meng, Susan J. Kimber

**Affiliations:** Faculty of Biology, Division of Cell Matrix Biology and Regenerative Medicine, School of Biological Sciences, Medicine and Health, University of Manchester, Manchester, United Kingdom; Università degli Studi della Campania, ITALY

## Abstract

Developmentally, the articular joints are derived from lateral plate (LP) mesoderm. However, no study has produced both LP derived prechondrocytes and preosteoblasts from human pluripotent stem cells (hPSC) through a common progenitor in a chemically defined manner. Differentiation of hPSCs through the authentic route, via an LP-osteochondral progenitor (OCP), may aid understanding of human cartilage development and the generation of effective cell therapies for osteoarthritis. We refined our existing chondrogenic protocol, incorporating knowledge from development and other studies to produce a LP-OCP from which prechondrocyte- and preosteoblast-like cells can be generated. Results show the formation of an OCP, which can be further driven to prechondrocytes and preosteoblasts. Prechondrocytes cultured in pellets produced cartilage like matrix with lacunae and superficial flattened cells expressing lubricin. Additionally, preosteoblasts were able to generate a mineralised structure. This protocol can therefore be used to investigate further cartilage development and in the development of joint cartilage for potential treatments.

## Introduction

Osteoarthritis (OA) is a painful degenerative disease affecting millions worldwide [1]. It is caused by the continued destruction of the articular cartilage, a tissue lining the ends of joints which enables their smooth movement, however, the underlying bone is also affected. This degeneration results in inflammation which further exacerbates the disease. Ultimately the effect of OA is pain for the sufferer, a reduction in mobility and an overall decrease in their quality of life, as well as great cost to society [2,3]. Articular cartilage has a low regenerative capacity and although artificial prostheses may be offered, a tissue engineered replacement of the damaged tissue involving cellular therapies is an attractive option. Human pluripotent stem cells (hPSCs) offer a great opportunity for the generation of cellular therapies for degenerative diseases. Their proliferative potential and ability to differentiate into any cell in the body [4] including chondrocytes for cartilage treatment, offers them distinct advantages over other cell types such as bone marrow mesenchymal stromal cells (MSCs), which, whilst able to produce chondrogenic cell types [5], are limited in expansion and therefore bulk supply of cells [6] and can have donor-specific issues with heterogeneity, quality and efficacy [7].

Importantly, in the majority of OA cases, both the articular cartilage and the underlying bone (forming the osteochondral system) are affected. Osteochondral constructs are therefore required to aid in the healing of such defects, while they are also likely to improve the integration of cartilage [8]. Consequently, a cell population able to produce both cartilage and bone of the articular joint (an”articular progenitor"), in an experimentally controlled manner, would be particularly useful for joint TE, to minimise protocol complexity and maximise healing potential.

Our group have developed directed differentiation protocols to produce prechondrocytes from hPSCs [9,10], with updated protocols replacing BMP4 with BMP2 to improve chondrogenesis [11], or substituting small molecules for growth factors to remove batch variability. However, though these differentiation pathways can produce prechondrocytes, they are not able to produce osteogenic cells, and only give moderate quality of cartilage pellets when cultured further [12]. One way of ensuring the production of cells present during the formation of the fetal tissue is to attempt to recapitulate the development process more closely [13]. The differentiation of hPSCs to chondrocytes has, to date, relied heavily on differentiation through the paraxial mesoderm pathway, generating chondrocytes able to produce recognizable cartilage like matrix [14–17]. Whilst chondrocytes can be derived through the paraxial mesoderm/ somite pathway, these cells do not typically populate the limb bud to generate the joints and their articular cartilage. As such, utilising this route may result in a different chondrocyte phenotype, for example expressing type-X collagen and high levels of RUNX2 indicative of mineralizing prehypertrophic non-hyaline cartilage [15]. Indeed some hPSC differentiation protocols rely on minimal use of differentiating reagents which would direct a more homogenous differentiating population and instead isolate the appropriately differentiated cells [18,19]. Differentiation protocols have also been reported through which preosteoblast have been derived, however these too required purification techniques to produce an enriched population [20,21].

Cells which produce the cartilage and bone comprising the joints, originate from the limb-bud, which develops from lateral plate mesodermal tissues [22]. Unlike paraxial somite derived cartilage in the body, this developmental pathway gives rise to the unique niche from which the long bone articular cartilage arises [23]. As such, this differentiation pathway should give rise to a stable hyaline cartilage phenotype, and likely generates chondrocytes with differences to those produced by the paraxial route [24] which gives the axial skeleton in development. Though there have been attempts to generate chondrocytes from cells deriving from lateral plate [25,26] they use undefined methods, such as formation of embryoid bodies rather than utilising directed differentiation [13]. More recently Loh *et al* have detailed the generation of lateral plate mesoderm derivatives from hPSCs, with indications of limb bud tissue [27]. This has been further built on by Yamada to produce chondrocyte-like cells, confirming the feasibility of this developmental route from hPSCs [28]. Building on our previous protocols and this additional knowledge, our study aims to recapitulate the developmental system of limb-bud formation to produce an osteochondral progenitor, which can give rise to both prechondrocytes and preosteoblasts in a directed manner. To this end we report a serum free **R**efined **A**rticular **P**rogenitor **I**ntegrated **D**ifferentiation (RAPID) protocol.

## Materials and methods

### hPSC cell culture

For continued pluripotent culture, human embryonic stem cells (hESC) lines Man-13 [29] and Man-7 [11] were cultured in feeder-free culture conditions in 6-well plates coated with vitronectin (5 ug/mL) (Thermo) in mTeSR1 (Stem Cell Technologies). Cells were passaged at 70–80% confluence with 0.5 mM EDTA supplemented with Revitacell (Gibco). Medium was initially changed after 24 hours to remove Revitacell, then subsequently every 2 days. The derivation of hESC cell lines was approved and licenced by the UK Human Fertilisation and Embryo Authority (licence R0171). Lines were derived with informed written consent of donors.

## RAPID progenitor differentiation protocol

**Fig 1:**
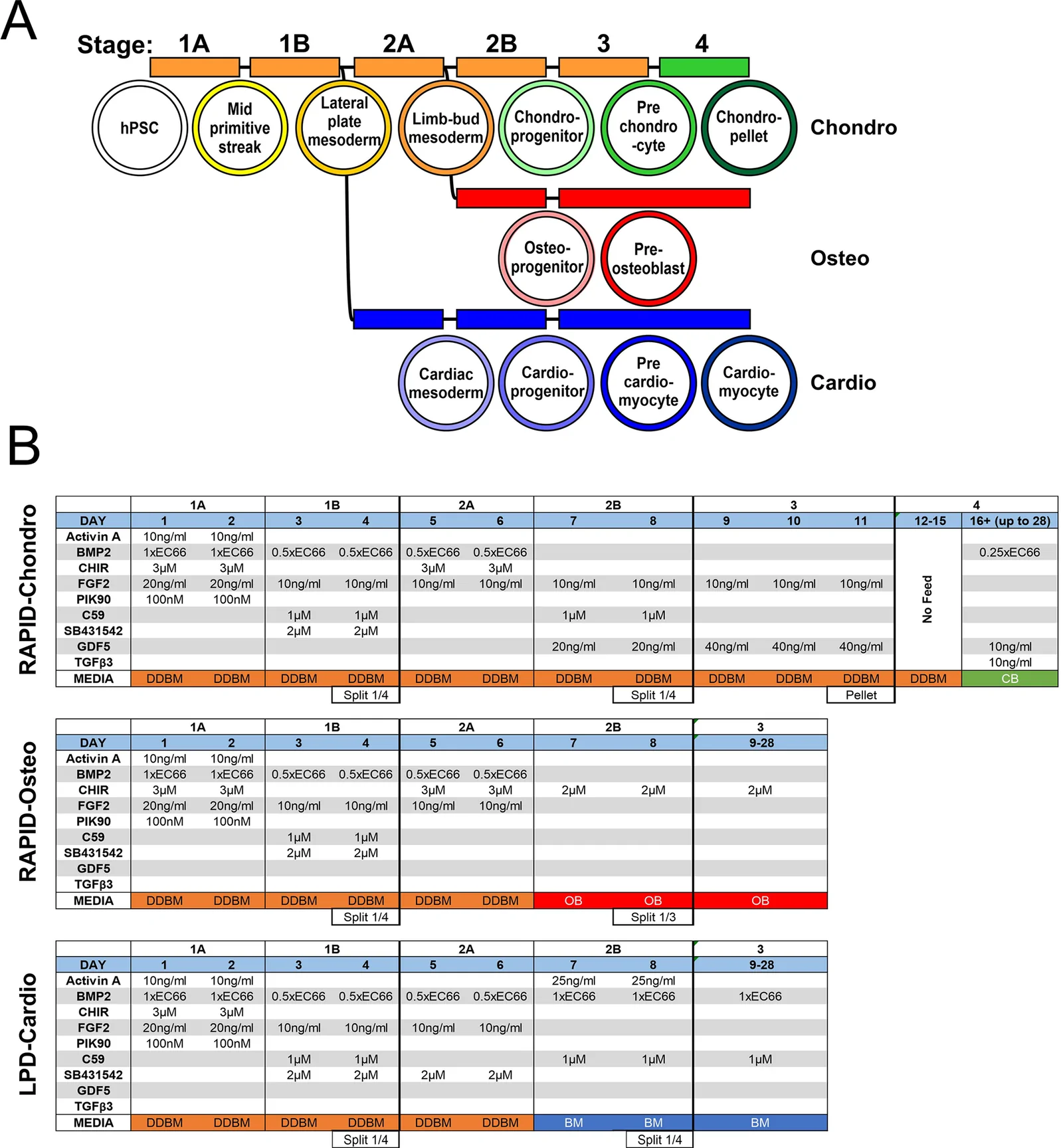
RAPID protocol. A) Flow diagram of stages and media required to achieve all 3 cell types during the RAPID protocol (including LPD). B) Growth factor regimen for RAPID (including LPD) protocols at each day for the chondrogenic, osteogenic and cardiogenic protocols.

hPSCs were split onto fibronectin coated (16.6 ug/mL) (Millipore) 6-well plates at 100,000 cells/cm^2^ and cultured till 60–70% confluence before the start of each protocol. Upon reaching confluence, pluripotency medium was removed, and directed differentiation basal medium (DDBM)(see S1 Data) added subject to particular differentiation pathways as described in full in Fig 1A. Briefly, for osteochondral lineages, cells were directed using our Refined Articular Progenitor Integrated Differentiation (RAPID) protocol, at first culturing cells in CHIR99021 (3mM) (Tocris), Activin A (1ng/mL) (Peprotech), BMP2 (R&D systems) (as BMP concentration is crucial in lateral plate development, and to avoid batch variation, each BMP batch activity was titrated and used at 66% of maximal effective concentration [EC66]), FGF2 (20ng/mL) (Peprotech) and PI3K inhibitor; PIK90 (100nM) (Selleckchem) to generate a culture expressing mid primitive streak markers (days 1–2) [27,30]. At day 3 only the growth factors BMP2 (EC66) and FGF2 (10ng/mL) were continued but with the addition of WNT inhibitor C59 (100nM) (Abcam) and ALK5 inhibitor SB431542 (2mM) (Tocris), to drive cells towards lateral plate mesoderm (days 3–4 orange lineage Fig 1A). At days 5–6, cultures were subjected to a CHIR99021 pulse to induce limb-bud-like mesoderm or withheld to promote cardiac-like mesoderm (blue lineage Fig 1A). Limb-bud mesoderm progenitors were further induced either through application of DDBM medium containing GDF5 (40ng/mL) (Peprotech) and BMP2 (0.5xEC66) to prechondrocyte cells (day 7–11, green lineage Fig 1A), or using osteogenic-basal medium (OM) (see S1 Data) containing CHIR99021 (2mM) to preosteoblast cells (days 7–8, red lineage Fig 1A) (7–8). Separately, day 5 lateral plate mesoderm progenitors were further differentiated to cardiac mesoderm and into cardiomyocytes through days 8–28 using Basal-cardio Medium (BM) (lateral plate derived [LPD] cardiac protocol, blue lineage Fig 1A). For the full application sequence of growth factors and small molecule see Fig 1B. For media compositions see S1 Data.

### Chondrogenic pellet culture

Day 11 prechondrocytes were removed from tissue culture plastic and centrifuged in 14ml polypropylene tubes at 300 xRCF in day 11 medium for 5 minutes to sediment cells. Caps were left loose to enable gaseous transfer and tubes incubated for 3 days at 37°C to facilitate the production of a spherical pellet, as detailed previously [12]. At day 11+3, medium was changed for chondrogenic-basal medium (CM) containing GDF5 (10 ng/mL), TGFβ3 (10 ng/mL) (Peprotech) and BMP2 (0.5x EC66). For full composition of media see S1 Data. Pellets were cultured for up to 28 days with medium changed every 3–4 days. Human articular chondrocytes were kindly donated by Professor Leela Biant’s lab and retrieved from non-diseased cartilage tissue from patients with end-stage osteoarthritis undergoing knee replacements surgery. Samples collected with written consent under NHS REC Reference 13/EM/0388 (IRAS ID: 114697).

### Osteogenic culture

Day 8 osteoprogenitors were removed from fibronectin coated plastic and split 1:3 into non-coated 6-well plates. Cells were cultured with OM supplemented with 2μM CHIR99021 changed every 3–4 days up to day 28 for QRT-PCR analysis or for matrix staining.

### QRT-PCR Gene transcriptional analysis

Samples were taken from both 2D and 3D culture as described previously [12]. Samples were transferred to RLT buffer directly and RNA extracted by use of a RNeasy QiAgen kit. Extracted RNA was converted to cDNA using the ABI-RT kit (Life technologies). Quantitative real-time polymerase chain reaction (qRT-PCR) was conducted using PowerUp™ SYBR™ green (Life technologies) and assayed for genes associated with stages of development and eventual cell types (Chondrogenic, osteogenic, cardiogenic) (see S1 Data for full primer sequences). Data was analysed and displayed as expression relative to housekeeping gene *GAPDH*.

### RNA sequencing and analysis

RNA samples were recovered from the pluripotent cells and day 11 prechondrocytes using RNAeasy kit. Samples were run on an Illumina HiSeq4000 sequencer. Unmapped paired-end sequences from the Illumina HiSeq4000 sequencer were tested by FastQC (http://www.bioinformatics.babraham.ac.uk/projects/fastqc/). Sequence adapters were removed, and reads were quality trimmed using Trimmomatic_0.39 (PMID: 24695404). The reads were mapped against the reference human genome (hg38) and counts per gene were calculated using annotation from GENCODE 36 (http://www.gencodegenes.org/) using STAR_2.7.7a (PMID: 23104886). Normalisation, Principal Component Analysis, and differential expression was calculated with DESeq2_1.28.1 (PMID:25516281). Functional analysis using Gene Ontology Biological Process (2018) and Reactome (2016) databases was performed with enrichR_3.0 (PMID: 23586463). The top 500 genes ranked by p-value were used as the genelist for these analyses. Visual analysis was generated using PRISM Graph-pad 9. Raw data were deposited in EMBL-EBI Array Express (accession number E-MTAB-11758).

### Alizarin red staining

Monolayer samples were washed in PBS, fixed in 4% PFA solution for 10 minutes at room temperature, then washed 3 times in deionized water. A 1% (W/V) Alizarin solution was added to the wells and incubated at room temperature for 30 minutes, then washed in deionized water until all excess dye had been removed. Samples were then air dried before images were taken under bright field using a vertically mounted camera (Nikon D330). Quantification was calculated as percentage area stained and performed using ImageJ. Briefly, stained areas were determined using a colour threshold level to select ‘red’ hues. The selected, stained area was then measured and percentage of total well area stained calculated.

### BCIP/NBT active ALP enzymatic stain

Samples were fixed in 4% PFA at room temperature for 10 minutes, then washed 3 times in PBS. Sufficient BCIP-NBT (Sigma, B5655) was added to cover the cell monolayer and incubated at 37°C for 20 minutes. The solution was removed then the monolayer was washed with deionized water 3 times and allowed to dry. Images were taken under bright field using a vertically mounted camera (as above). Quantification was performed using ImageJ as described above, but ‘blue’ hues were used as a colour threshold.

### Western blotting

Cells were initially lysed in RIPA buffer (Sigma, R0278) with complete protease inhibitor ULTRA tablets (Roche, 589297001) and protein concentration quantified using a Pierce™ BCA assay (Thermo, 23252). A mixture containing 30μg protein and 1X Pierce™ Lane Marker Reducing buffer (Thermo, 39000) was then incubated at 96°C for 10 minutes. Samples were then loaded onto a NuPAGE™ 10% Bis-Tris Protein Gel (Thermo, NP0301BOX) alongside a pre-stained protein standard ladder (NeB, P7719). Protein bands were transferred to a nitrocellulose membrane (Thermo, IB23001) before blocking with 5% Milk-PBS-Tween (Sigma, PP9416). Membranes were then incubated with a SOX9 primary antibody (Protein Tech, 67439) or GAPDH (Cell Signalling Technology, 2118) at 1:1000 in Milk-PBS-Tween overnight at 4°C. Membranes were then washed 3 times with PBS-Tween for 10 minutes at room temperature. The secondary antibody (IRDye® 680RD Goat anti-mouse, LI-COR, 925–68070 or IRDye® 800CW Goat anti-rabbit, LI-COR, 925–68070) was then added at 1:15,000 in Milk-PBS-Tween and incubated for 1 hour at room temperature. Membranes were washed and scanned with an Odyssey® CLX Infrared Imaging System (LI-COR). Bands were quantified with ImageJ and SOX9 expression normalised to GAPDH.

### Histology

Chondrogenic pellets were fixed in 4% PFA overnight at 4°C then transferred to 70% ethanol before processing and embedding in paraffin-paraformaldehyde blocks. Embedded samples were sectioned at 5μm thickness. Sections were stained for H&E, Picrosirius red or Alcian blue.

For immunohistochemistry, sections were subject to 0.1M citrate antigen retrieval at 95°C for 10 mins before the primary antibody for type-II collagen (Mouse, Millipore, MAB8887), aggrecan (Rabbit) [31], lubricin (Rabbit, Abcam, ab28484), or type-I collagen (Rabbit, Gentour, OARA02579) was added. For IgG controls, IgG anti-mouse (Cell Signaling, 3900s) and IgG anti-rabbit (Cell Signaling, 5415s) were used. Samples were incubated overnight at 4°C, then treated with either fluorescent secondary antibodies (Thermo) and ProLong mountant (Thermo) for visualisation under a microscope (Olympus IX71), or with a biotin conjugated secondary antibody (R&D) and visualised using, streptavidin peroxidase and 3,3′-Diaminobenzidine (DAB). Images were taken on a slide scanner (3D Histech Panoramic250), or EVOS M7000 (Invitrogen).

### Statistical analysis

All statistical analysis was run using Prism Graph-pad. Samples were tested for normality using a D’Agostino-Pearson test. Statistical significance was calculated using Kruskal-Wallis (non-parametric ANOVA), Mann-Whitney (non-parametric non paired) or Wilcoxen (non-parametric paired significance test) (indicated in figure legend). A p-value of ≤ 0.05 was considered as statistically significant.

## Results

We set out to generate prechondrocytes which would develop further to form joint-like cartilage though the lateral plate mesoderm route. We named the developed protocol Refined Articular Progenitor Integrated Differentiation (RAPID) protocol and checked its authenticity by inducing other lateral plate derived differentiation derivatives from early-stage cells.

### The generation of osteochondral progenitors through lateral plate mesoderm

**Fig 2:**
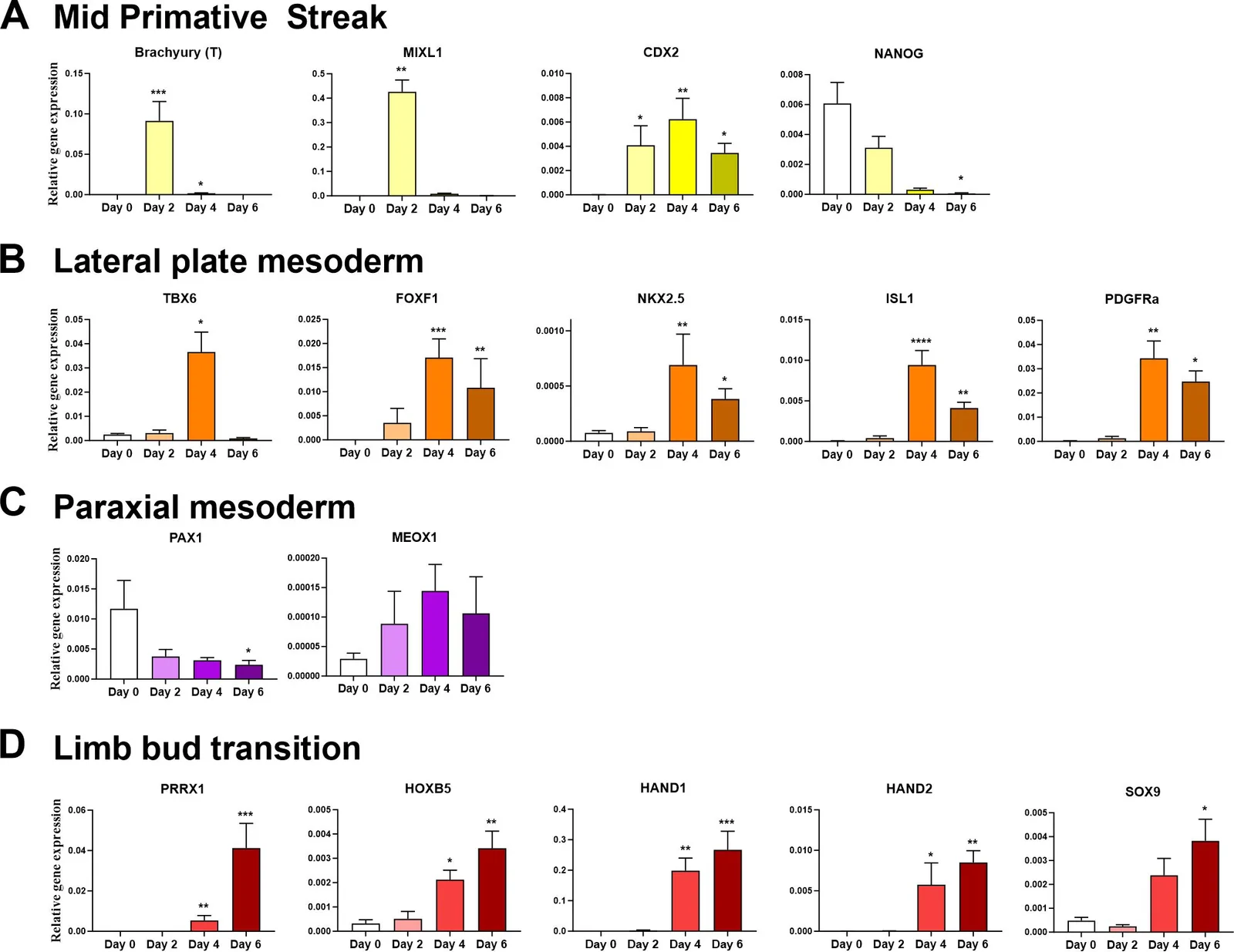
hPSCs are directed through lateral plate lineage to limb-bud like progenitor cells. Gene expression relative to *GAPDH* using 2^-Δct^ +SEM, for transcriptional analysis of hPSCs differentiating into A) mid-primitive streak (Day2), B) lateral plate mesoderm (Day4), C) paraxial markers and D) limb-bud transition like cells (Day 6). D’Agostino-Pearson test was used to test for normality. All series tested using Kruskal-Wallis test to determine statistical significance. Significance relative to hPSCs (day 0) (* ≤0.05, **≤0.01, ***≤0.001, ****≤0.0001). (N = 6 independent experiments).

In the first step ActivinA, FGF2, BMP2 and the GSK3 inhibitor CHIR99021 (to replace Wnt3a) were used to generate mid-primitive streak like cells as employed previously by ourselves [12] and others [27]. Additionally, the PI3K inhibitor PIK90 was used to help inhibit endodermal differentiation [32]. QRT-PCR gene transcriptional analysis indicated a clear increase in the primitive streak associated genes Brachyury (T) [33] and *MIXL1* [34], and mid/posterior primitive streak *CDX2* was present at day 2 of the differentiation protocol (Fig 2A) [35]. Following this and the removal of ActivinA and CHIR99021, *CDX2* expression was present during cell maturation towards mesodermal tissue, together with high *HAND1* and *HAND2* expression indicating lateral plate specification [27]. Additionally, transient *TBX6* transcripts were observed during the differentiation to mesodermal tissue [36] (Fig 2B). *TBX6* subsequently diminished at day 6 as expected. Importantly the transcription factors *FOXF1*, *NKX2*.*5* and *ISL1* were also significantly upregulated by day 4. These, together with *CDX2*, are essential for lateral plate mesoderm specification [35,37]. During the mesodermal and subsequent stages there was also no significant increase in the paraxial mesoderm associated marker *MEOX1*, and *PAX1* was significantly decreased by day 6, indicating significant paraxial mesoderm derived populations were unlikely to be differentiating (Fig 2C).

Following a second application of CHIR99021 (day 5–6) on day 6, there was a decrease in the expression of lateral plate mesoderm markers (Fig 2B), which coincided with an increase in limb-bud specification markers *PRRX1*, *HOXB5*, *HAND1* and *HAND2* as well as *SOX9* (Fig 2D). Additionally, the pluripotency/primitive steak marker *NANOG* was significantly decreased by this stage (Fig 2A). These day 6 cells expressed *PRRX1*, *PDGFRa* and *SOX9* indicating specification to an osteochondral progenitor (OCP) [22,23], and were then subsequently used for further chondrogenic or osteogenic differentiation.

### Transition from limb-bud like osteochondral progenitors to prechondrocytes

**Fig 3:**
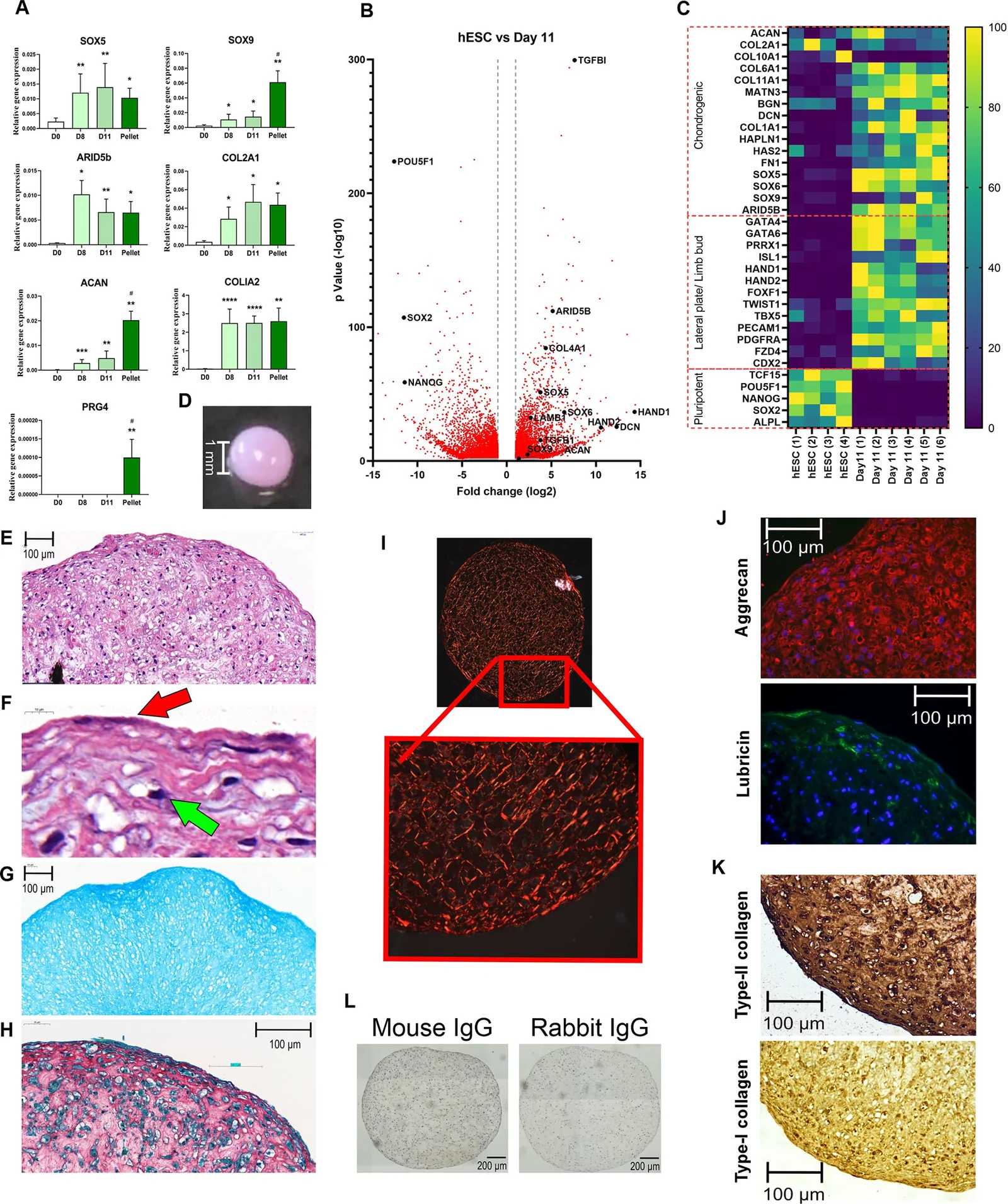
Differentiation of chondroprogenitors through to developing chondrocytes, from limb-bud like progenitor cells. A) QRT-PCR analysis of chondrogenic gene expression during differentiation in 2D (until day 11) and in 3D pellet culture (day 11+14). Gene expression data displayed as relative to housekeeping gene *GAPDH* +SEM. Mann-Whitney test was used to determine statistical significance relative to hPSCs (day 0) (+ ≤0.05, ++≤0.01, +++≤0.001). # indicates significant difference to day 11 (N = 5 independent experiments). B) Volcano plot for differential gene expression in day 11 cells (N = 3 independent experiments with duplicate samples) compared to pluripotent hESCs (N = 4 independent experiments). Significant gene changes detected with log2 fold change (log2FC) ≥1 or log2FC ≤-1, with p-value ≤0.05. Red plots indicate adjusted p-value ≤0.05. Targets of interest are indicated in black and named. C) Heat map of normalised gene expression for target pluripotent, limb bud and chondrogenic gene expression targets in pluripotent (N = 4 independent experiments) and day 11 cells (N = 3 independent experiments with duplicate samples). Data displayed as percentage normalised to lowest and highest expressed for each gene of interest. D) Image at day 11(2D) +28 d in 3D for pellet. E) H&E histological stain of day 11+28 pellet, and F) enlarged view of pellet periphery in H&E including arrows indicating flattened surface cells (red arrow) and cells in lacune (green arrow). G) Alcian blue stain of day11+28 pellet. H) Picrosirius red collagen matrix stain. I) Polarised light imaging of Picrosirius red staining of day 11+28 pellet. J) Immunofluorescence images for day 11+28 pellets stained for Aggrecan (red), Lubricin (green) and K) immunohistochemistry for type-II collagen and type-I collagen (IHC). In J) DAPI and in K) haematoxylin were used for visualisation of cell nuclei. L) IgG controls for mouse and rabbit primary antibodies.

The lateral plate derived limb-bud like cells were driven in their differentiation to chondrogenic progenitors using inhibition of the Wnt pathway with C59 and the addition of the articular joint growth factor GDF5. At day 8 chondroprogenitor cells expressed significant levels of the transcription factors *SOX5*, *SOX9* and *ARID5B* as well as the ECM components *ACAN*, *COL2A1* and *COL1A2* compared to day 0 (Fig 3A). Development of cells continued with additional GDF5 till day 11 and resulted in sustained expression of the chondrogenic markers, however *PRG4* (lubricin) was not expressed at either day. RNAseq of the day 11 cells supported QRT-PCR gene expression data and indicated significant increases in limb bud and chondrogenic transcription factor and ECM genes and a decrease in expression of pluripotency-associated genes (Fig 3B). This included non-traditional markers such as *TGFBi*, which has been shown to be important in chondrocyte development [38], and which was highly significant in our data-set. Heat map analysis indicates the expression of lateral plate/ limb bud genes as well as the start of chondrogenic associated gene expression in these cells (Fig 3C), with GO term and Reactome again indicating a change in ECM organization and collagen fibril assembly, and a shift in pluripotency associated gene expression (S1A Fig). These prechondrocytes were then taken into pellet culture to test their chondrogenic differentiation ability.

A further hPSC line, Man7, was subjected to chondrogenic differentiation using the RAPID protocol, and also displayed significant expression of the chondrogenic markers *SOX5*, *SOX9*, *ARID5b*, *COL2A1*, and *ACAN* as well as *COL1A2* at day 11 (S1B Fig).

### Prechondrocytes can form cartilage-like matrix

To test the chondrogenic potential of the prechondrocytes, day 11 cells were pelleted and cultured for up to 28 days. Whilst cells from day 8 of the differentiation protocol can be used to produce pellets, in our experience those pellets are fragile, tend to disintegrate after a few days and are noticeably less stable than those produced from cells at day 11. Therefore, day 11 cells were used for the formation of pellets.

This further differentiation in 3D pellet culture resulted in the continued high-level expression of *SOX5*, *ARID5B*, *COL2A1* and *COL1A2*, with significant increases in *SOX9* and *ACAN* compared to day 11. Importantly, in 3D pellet culture cells also expressed the articular surface proteoglycan *PRG4* (lubricin) (Fig 3A). Though the pellets contain developing chondrocytes, the cells were compared to adult human articular chondrocytes obtained at passage 1, and displayed similar gene expression for *SOX9* and *ACAN*, though *COL2A1* expression was lower in the pellets (S1C Fig). *COLX* gene expression was below detectable levels (after 40 cycles of PCR) during 2D and 3D pellet culture, indicating the cells were not developing into pre-hypertrophic chondrocytes. This lack of type-X collagen gene expression was supported by RNAseq, in which it had a maximum copy number of 1, seen in the day 11 prechondrocytes.

After 28 days in 3D pellet culture, pellets displayed a spherical, translucent, glossy appearance similar to the articular cartilage surface (Fig 3D). Histological staining displayed a cartilage-like structure (Fig 3E) with cells present in lacunae (Fig 3F, green arrow on insert), and surface cells showing a flattened elongated morphology (Fig 3F, red arrow). The pellets stained for Alcian blue, with a stronger intensity towards the surface (Fig 3G), whilst Picrosirius red staining indicated a dense collagen matrix (Fig 3H). Indeed, polarised light imaging after Picrosirius red staining indicated fibrillar collagen networks throughout the pellets, with a change in orientation towards the surface of the pellets (Fig 3I), reminiscent of the expected change in cell alignment at the cartilage surface. Histological analysis of these pellets identified the key cartilage components aggrecan, lubricin and type-II collagen within the structure (Fig 3J). Whilst aggrecan was present throughout the pellet structure, lubricin was only present near the surface of the pellet. Additionally, type-II and type-I collagen staining was present in the pellet. Moreover, type-II collagen staining was strong throughout the structure, with darker staining towards the surface which was not evident with type-Istaining for which was lower (Fig 3K).

### Transition from limb-bud-like osteochondral progenitors to preosteoblasts

**Fig 4:**
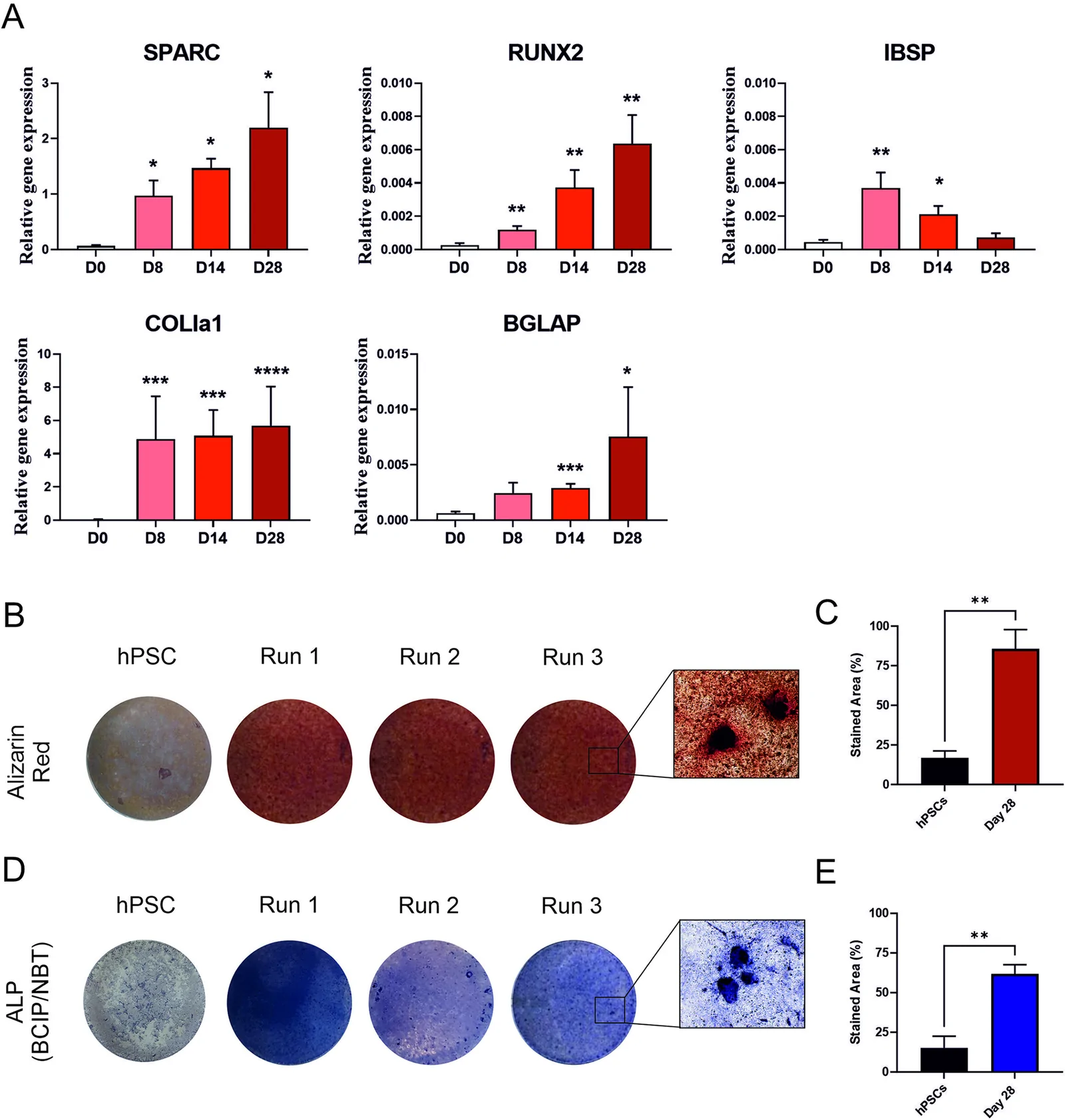
Differentiation of preosteoblast like cells from limb-bud-like progenitor cells. A) QRT-PCR gene expression analysis during osteogenic differentiation pathway up to day 28. Gene expression data displayed relative to housekeeping gene *GAPDH* +SEM. Mann-Whitney test was used to determine statistical significance. Significance relative to hPSCs (day 0) (* ≤0.05, **≤0.01, ***≤0.001) (N = 5 independent experiments day 0 to 14, N = 3 independent experiments for day 28). B) Alizarin Red staining for mineralisation assay of undifferentiated hPSCs (Day 0) and preosteoblast-like cells at the end of differentiation (Day 28) (N = 3 independent experiments). C) Quantification of stained area positive for Alizarin Red at day 0 and day 28 (percentage total area stained). Data presented as mean percentage area + SEM. Unpaired t-test was used to determine statistical significance (** p<0.01) D) BCIP-NBT active ALP enzyme assay for preosteoblast like cells cultured till day 28 (N = 3 independent experiments). E) Quantification of stained area positive for BCIP-NBT at day 0 and day 28 of differentiation. Data presented as mean percentage area + SEM. Unpaired t-test was used to determine statistical significance (** p<0.01).

To assess the osteogenic potential of the limb-bud-like progenitor cells, differentiation was continued after day 6 of the above protocol in osteogenic medium. Gene expression analysis showed significant increases in expression of the early osteogenic transcription factors *SPARC* (osteonectin), *IBSP* and *RUNX2* by day 8 in comparison to day 0 (Fig 4A). This significant increase was sustained at day 14. The essential bone ECM component type-I collagen was also significantly increased in gene expression by day 8 and remained high at day 14, with significant expression of the osteoblast-expressed ECM component, *BGLAP* (osteocalcin), by day 14. No *COLX* was detected at any stage. Differentiating preosteoblast cells were cultured till day 28 and showed continued significant expression of osteoblast markers compared to day 0, however *IBSP* had fallen by this time point. Additionally, their matrix mineralisation ability was assessed by staining of the resulting tissue with Alizarin red, which resulted in an orange/red colouring of the produced matrix (Fig 4B and 4C) including nodes apparent in the matrix (insert). This indicated the formation of inorganic calcium rich deposits. BCIP-NBT staining produced a strong blue colour throughout the matrix, suggesting active ALP enzyme activity in the matrix/cells (Fig 4D and 4E).

### Cardiomyocyte derivation from lateral plate mesodermal cells

**Fig 5:**
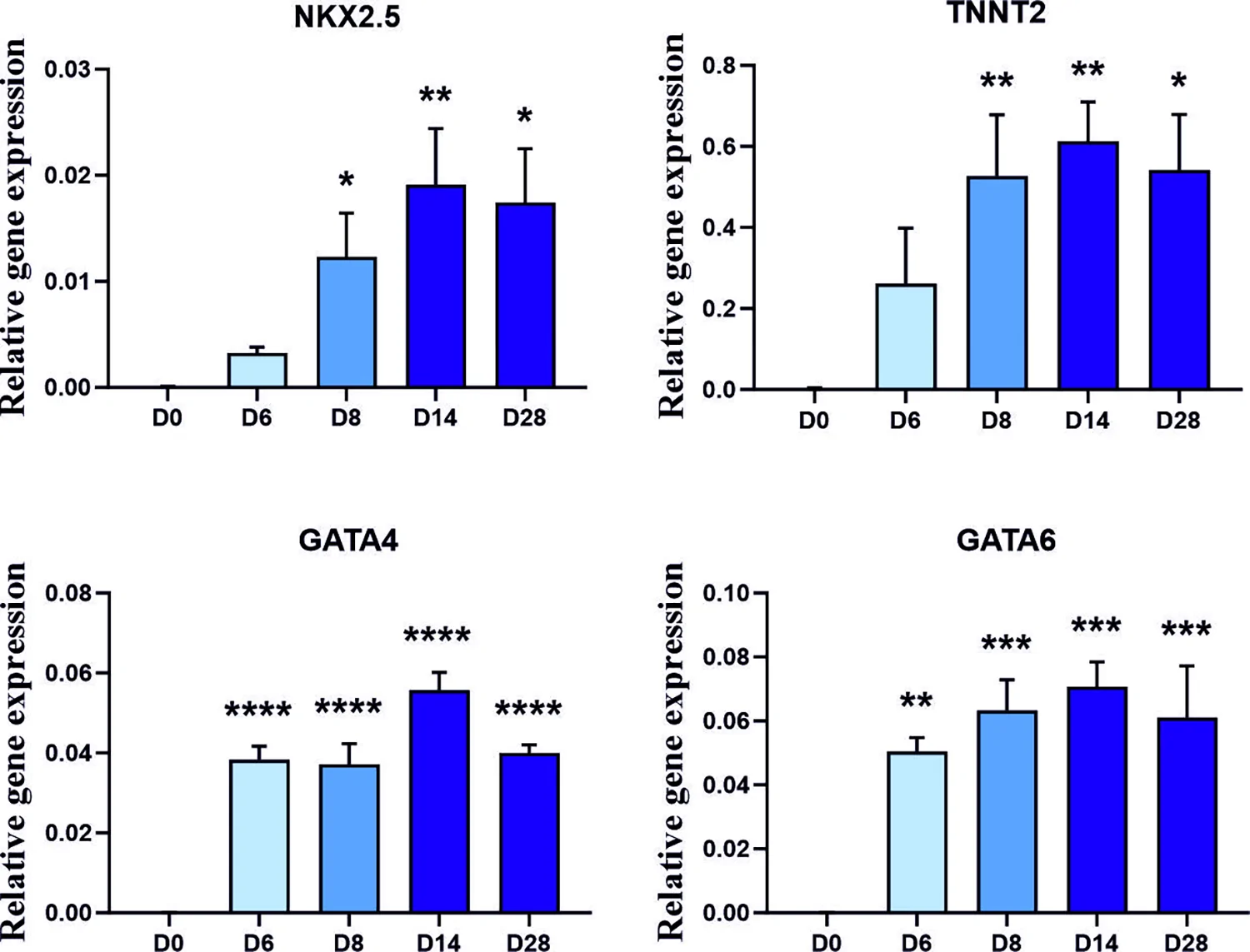
Differentiation of cardiomyocyte progenitor cells from lateral plate lineage through cardiac mesoderm. Gene transcriptional analysis of cardiomyocyte genes during differentiation to beating cardiomyocytes. (For video of contracting tissue please see supplementary evidence). Gene expression data displayed relative to housekeeping gene *GAPDH* +SEM. Mann-Whitney test was used to determine statistical significance. Significance relative to hPSCs (day 0) (+ ≤0.05, ++≤0.01) (N = 5 independent experiments for days 0 to 14, N = 4 for day 28).

To confirm our route to chondrocytes and osteoblasts we tested if cardiomyocyte progenitors could be generated from day 4 lateral plate-like cells (Fig 1) as cardiomyocytes also originate from the lateral plate mesoderm. Instead of promoting a limb-bud like cell, day 4 lateral plate-like cells were cultured on, without a CHIR99021 pulse at day 5–6, to generate a cardiac-like mesoderm. The resulting day 6 cells were then transferred to a cardiac mesoderm differentiation cocktail in BM for up to 28 days (Fig 1A). Gene transcription analysis of day 6 samples showed a significant increase in the cardiac marker *NKX2*.*5*, the cardiac specific *TNNT2* transcript, as well as cardiac maturation transcription factors *GATA4* and *6* [39] (Fig 5). These cardiac markers remained significant up to day 28 (Fig 5). Cells formed clusters around day 11, which began beating asynchronously (S1, S2 and S3 Videos). By day 28 these clusters had increased in size and were more synchronous in their beating.

### RAPID Protocol shows improved chondrogenic gene expression to standard DDP

**Fig 6:**
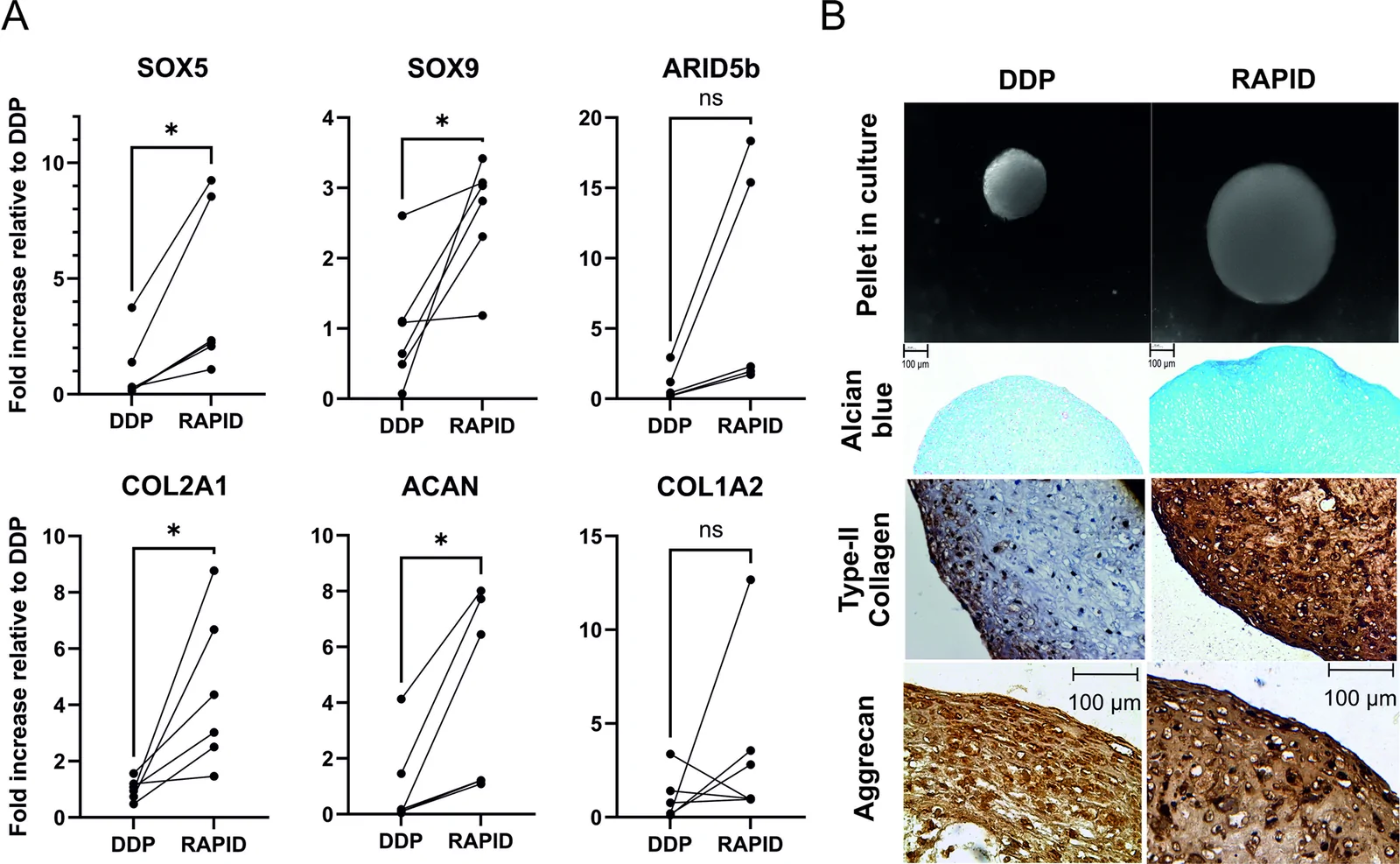
Comparison in chondrogenic gene expression between paired existing DDP and lateral plate derived RAPID cells. Man7 PSCs were differentiated to prechondrocytes through both the DDP and RAPID protocols. A) QRT-PCR gene expression assessment for the main transcription factors and ECM components in pair match DDP and RAPID samples. DDP samples were taken at day 14 compared to day 11 in the RAPID protocol. Data is expressed as normalised to existing DDP protocol, with connecting lines indicating paired experiments. B) Light microscopy image of pellets, Alcian blue matrix staining, immunohistochemistry for type-II collagen and aggrecan in 2D+28 day pellets. A Wilcoxen test was used to determine statistical significance. + indicates significance to hPSCs (day 0) (+ ≤0.05, ++≤0.01) (N = 6 independent experiments).

We tested the chondrogenic potential of the lateral plate derived RAPID prechondrocytes compared to those of the original defined differentiation protocol (DDP) [7]. Paired RAPID and DDP samples were differentiated and the expression of the key chondrogenic factors were compared at the end of the respective 2D protocols. Day 11 RAPID prechondrocytes showed increased gene expression compared to Day 14 DDP cells, with significant increases in transcription factors *SOX5* and *SOX9* as well as the matrix components *COL2A1* and *ACAN* (Fig 6A). Additionally, we also compared the histology of pellets produced from the DDP [12] with those of the RAPID prechondrocytes. RAPID prechondrocyte- derived pellets are considerably larger than those from DDP and gave a deeper Alcian blue staining (Fig 6B). Whilst both pellet types stained for the main matrix components, the RAPID pellets displayed greater staining for type-II collagen and aggrecan, and maintained a stronger type-II collagen stain towards the pellet surface.

## Discussion

In this study we have detailed the use of an improved, defined protocol (RAPID), to generate lateral plate derived prechondrocytes, preosteoblasts and cardiomyocytes from hPSC. The protocol relies on knowledge of development; differentiating the cells first through mid-primitive streak, then lateral plate mesoderm from which limb bud OCPs can be derived, and then into both prechondrocytes and preosteoblasts. Furthermore, beating cardiomyocyte-like cells can be derived from a generated cardiac mesoderm intermediate.

Though other groups have reported the production of lateral plate mesoderm from hPSCs [27,28], their focus has been on specifying cardiac progenitors rather than limb-bud derived tissues such as osteochondral progenitors, or through isolating subpopulations. Others achieved articular chondrogenesis through the use of EB differentiation [26,40,41]. Loh *et al* [27] detailed a fast pathway to lateral plate and limb bud, which wasextended by others [28]. However, the dual chondrogenic and osteogenic lineage differentiation of these cells was not studied.

We modified our previous defined differentiation protocol (DDP) [9,11,12] influenced by the invaluable early lineage data of Loh *et al* [27] and others [35], and report growth factor and small molecule requirements for production of these cell types from the Man l3 and 7 lines without need for selection procedures. The time-specific growth factor applications was also influenced by protocols for developmentally adjacent mesoderm lineages such as kidney [30], which we have previously utilised to generate kidney organoids [42]. As in our original differentiation pathway, these protocols all indicate the importance of early application of ActivinA, FGF, and CHIR99021 as surrogate for Wnt stimulation. We utilised BMP2 in our protocol, having previously identified it as efficient in inducing hPSC-chondrogenic gene expression [11], maintaining this throughout the protocol alongside a CHIR pulse at days 5–6, to form OCPs. Importantly, we evaluated the activity of BMP2 required in our differentiation protocols. Use of BMP *activity* makes our protocols more reproducible to reduce the issue of batch variation in such activity as derivation of lateral plate mesoderm requires a careful balance of BMP signalling.

Using this protocol we can produce articular OCPs in as little as 6 days, with cells expressing *PRRX1*, *SOX9* and *PDGFRa* as in the developing limb [22,23]. To achieve articular joint-like prechondrocytes, between day 8 and 11, we utilised GDF5, a critical signalling molecule in the natural development of articular cartilage [43–46], which we used previously [11,12,47]. This ensured a robust stable structure, similar to our previous findings [11]. Importantly, OCPs did not express significant *PAX1* and *MEOX1* indicating they were not developing through a paraxial (somite) lineage, confirmed by RNAseq data at day 11. In paired samples, the RAPID protocol-produced prechondrocytes which expressed significant increases in the transcription factors *SOX5* and *SOX9*, as well as the matrix components *COL2A1* and *ACAN*. Though differentiation through paraxial mesoderm has frequently been used to produce chondrocytes [13], ultimately appropriate chondrogenic differentiation through the limb-bud-like mesoderm is likely to produce cells more akin to the native long bone articular chondrocytes developed from growth plate progenitors and interzone cells [43]. This unique route may affect the cells’ maturation and long-term phenotype, as long bone articular cartilage is not produced through paraxial-somites.

This current protocol is faster than our previous protocol [12], and generates pellets more similar to hyaline cartilage by following a lateral plate pathway. Crucially, pelleting led to the formation of smooth translucent tissue, which displayed cells in a lacuna structures. The pellets are larger, with more uniform outline and stronger Alcian blue, GAG, staining. As well as staining for the major chondrogenic matrix proteins, our RAPID protocol produced pellets stained for the surface zone proteoglycan lubricin (*PRG4*) [48,49] in the outer region. This together with a more flattened cell phenotype, a change in matrix orientation, and an increased fibrillar collagen content, indicates a similarity to the superficial zone in articular cartilage [50,51]. These observations suggest production of cartilage with distinct zones: crucial for cell engineering for replacement of damaged cartilage [48,50]. Type-X collagen (*COLX*) expressed in mineralising cartilage such as at the bone-interface was not evident in our system.

In addition to prechondrocytes, the OCPs differentiated separately into preosteoblasts in 14 days, with continued culture of these cells producing a moderately mineralised osteogenic matrix. Indeed osteogenic differentiation of hPSC has been achieved by many different groups [52–54] including through LP [20]. During the continued osteogenic culture of these cells, gene expression of *RUNX2*, *OCN*, and *BGLAP* increased. We saw a rise of *IBSP* (not previously reported in hESC-differentiation protocols) during early osteogenesis, but a fall during late mineralisation. Interestingly, the differentiation protocol of Tan *et al* [55], also employed by Kidwai *et al* [20] used BMP4 and VEGFa for lateral plate mesoderm differentiation, whilst our protocol utilised BMP2. However, they employed a cell sorting step to enrich a lateral plate population before continuing further differentiation.

In the current study, we used osteogenesis as proof of principal that the limb-bud progenitors are able to generate mineralizing immature bone-like tissue as well as an articular cartilage lineage (no *COLX*, but *PRG4*, *ACAN* and *COL2A1* expressing cells). In the long term this may allow development of osteochondral grafts produced from a unified differentiation process. Such a graft requires formation of a true osteochondral construct, complete with a mineralised cartilage boundary, which will ultimately allow for better integration of the graft and its long-term survival in situ. This strategy would reduce the complexity inherent in using multiple cell populations. Finally, whilst the RAPID protocol is designed to generate OCP and subsequently prechondrocytes and preosteoblasts, we also showed the capability to generate beating cardiomyocytes. Progression through cardiac mesoderm [27], by including BMP, as used by Takei *et al* [56], to generate beating cells confirmed the lateral plate lineage.

Whilst we were able to show the further differentiation of the OCP cells into chondrogenic- and osteogenic-like cell types, further work should now aim to demonstrate their ability to participate in repair of an osteochondral defect and integrate with host joint tissue.

In conclusion, we have demonstrated the fast and efficient production of limb-bud/ articular progenitors, which are able to form both cartilaginous and osteoid tissues with appropriate culture using a defined growth factor and small molecule regimen. The production of both cell types from the lateral plate lineage could be important in aiding graft incorporation by facilitating the formation of both chondral and subchondral tissue, and generating the appropriate matrix required for hyaline cartilage. It is now important, as with paraxial differentiation [15], to understand the integrated time course of transcriptome, epigenome and proteome changes which occur during this protocol., Indeed single cell/nuclear RNAseq would be invaluable to dissect out the likely subtly different cell types generated, which provide matrix distribution heterogeneity. Comparison of different protocols may indicate additional important factors associated with joint cartilage development.

## Supporting information

S1 Fig A) Biological process GO Term and Reactome enrichment charts for expression change in RNAseq analysis of day 11 prechondrocyte samples compared to hESCs. B) Differentiation of Man7 hPSCs to prechondrocytes. Man7 cells were differentiated through the RAPID protocol to produce prechondrocytes with samples taken at day 11 (D11) (N = 3 independent experiments with duplicate samples). Gene expression was assessed by qRT-PCR for chondrogenic genes. Mann-Whitney test was used to determine statistical significance. Gene expression data displayed relative to housekeeping gene *GAPDH* +SEM. Significance relative to hPSC (day 0) control cells (+ ≤0.05, ++≤0.01) (N = 6 independent experiments). C) Gene expression of RAPID protocol derived developing chondrocytes (N = 5 independent experiments) from day 11+28 pellets compared with RNA extracted from adult human articular chondrocytes (N = 3 individual biological samples). Gene expression data displayed relative to housekeeping gene *GAPDH* +SEM.
(TIF)

S2 Fig Protein analysis of SOX9 expression during RAPID-T differentiation. A) Western blot protein expression analysis of SOX9 (55kDa) and housekeeper GAPDH (37kDA) in pluripotent, Day 6 and Day 11 cells. B) Densitometry quantification of bands shown in A). SOX9 protein expression normalised to GAPDH. N = 1.
(TIF)

S1 Data Media compositions, RT-PCR primers. (DOCX)

S1 Video Beating muscle from day 11 cardiomyocyte cultures. (MP4)

S2 Video Beating muscle from day 11 cardiomyocyte cultures. (MP4)

S3 Video Beating muscle from day 11 cardiomyocyte cultures. (MP4)
