# Supplementary figures and images for: Directed differentiation of hPSCs through a simplified lateral plate mesoderm protocol for generation of articular cartilage progenitors
Source: PLoS One. 2023 Jan 27;18(1):e0280024. doi: 10.1371/journal.pone.0280024 (PMC9882893; doi:10.1371/journal.pone.0280024)

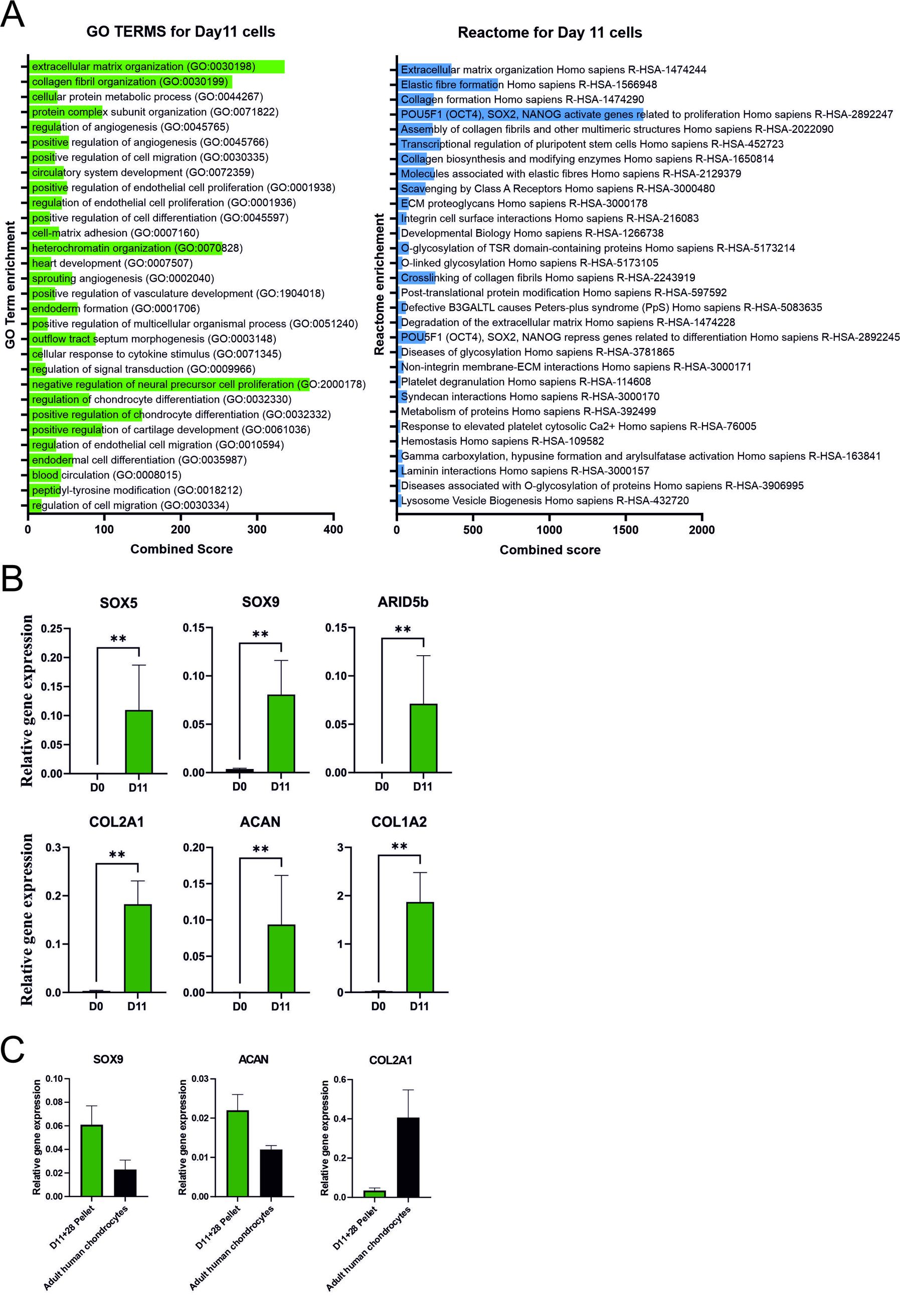

Supplement: S1 Fig — A) Biological process GO Term and Reactome enrichment charts for expression change in RNAseq analysis of day 11 prechondrocyte samples compared to hESCs. B) Differentiation of Man7 hPSCs to prechondrocytes. Man7 cells were differentiated through the RAPID protocol to produce prechondrocytes with samples taken at day 11 (D11) (N = 3 independent experiments with duplicate samples). Gene expression was assessed by qRT-PCR for chondrogenic genes. Mann-Whitney test was used to determine statistical significance. Gene expression data displayed relative to housekeeping gene GAPDH +SEM. Significance relative to hPSC (day 0) control cells (+ ≤0.05, ++≤0.01) (N = 6 independent experiments). C) Gene expression of RAPID protocol derived developing chondrocytes (N = 5 independent experiments) from day 11+28 pellets compared with RNA extracted from adult human articular chondrocytes (N = 3 individual biological samples). Gene expression data displayed relative to housekeeping gene GAPDH +SEM. (TIF) [file pone.0280024.s001.tif]

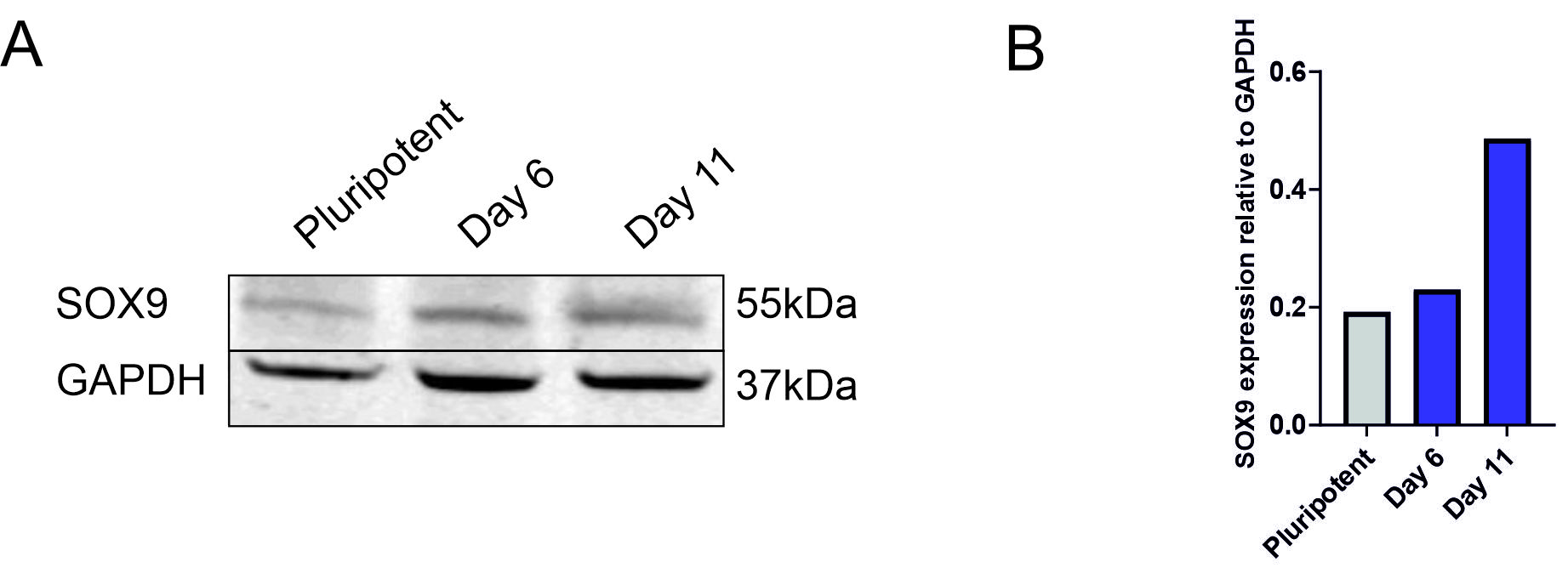

Supplement: S2 Fig — A) Western blot protein expression analysis of SOX9 (55kDa) and housekeeper GAPDH (37kDA) in pluripotent, Day 6 and Day 11 cells. B) Densitometry quantification of bands shown in A). SOX9 protein expression normalised to GAPDH. N = 1. (TIF) [file pone.0280024.s002.tif]
